# To eat or not to eat oats: factors associated with oats consumption using the I-Change model

**DOI:** 10.1186/s12889-024-20044-4

**Published:** 2024-11-19

**Authors:** S. Van Kol, H.C. van der Horst, H. de Vries

**Affiliations:** 1 Faculty Health, Medicine, and Life Sciences, Maastricht University, Maastricht, The Netherlands; 2 Keep Food Simple, Driebergen-Rijsenburg, The Netherlands; 3 CAPHRI School for Public Health and Primary Care, Faculty of Health, Medicine and Life Sciences, Maastricht University, P.O. Box 616, Maastricht, 6200 MD The Netherlands

**Keywords:** Consumer behaviour, Oats, Predisposing factors, Cognizance, Risk perception, Attitude, Social norms, Self-efficacy, The Integrated Change Model (ICM)

## Abstract

A suboptimal diet is responsible for more deaths worldwide than any other risk. A dietary factor that needs more attention is whole-grain consumption because of its proven health, nutritional, and environmental benefits. Despite these benefits, the daily whole-grain and dietary fibre intake by Dutch residents is low and the reasons for this low consumption are unknown. This study analysed the facilitating and hindering factors associated with (non) oat consumption using the Integrated Change Model (ICM). It compared which factors distinguished oat consumers from moderate and low-oat consumers using a cross-sectional, quantitative study design. The questionnaire used in the quantitative study was based on the results a qualitative study that validated the findings identified in the literature and previous ICM studies. Data was collected using an online survey (Qualtrics). In both studies, the sample of participants included individuals age > 18, of both genders residents in the Netherlands without an allergy to oats, and who read or speak fluently Dutch or English. In total, 299 residents were included in the study. The results indicated that non-oat consumers were less health-conscious, had limited awareness of their dietary behaviours, possessed less knowledge, and were less aware of the severity and susceptibility to various health conditions, such as high cholesterol. They also received fewer prompts to consume oats, were less convinced of the rational and emotional benefits of oats, experienced less social support, reported more obstacles, and had lower self-efficacy regarding oat consumption. The Integrated Change Model (ICM) explained 55% of the variance in intention and 38% in behaviour. Consequently, efforts to promote oat consumption among Dutch adults should address these factors to enhance awareness, increase motivation, and facilitate actions to boost oat intake.

**Supplementary Information:**

The online version contains supplementary material available at 10.1186/s12889-024-20044-4.

## Introduction

Numerous studies highlight the need for human health to change and improve their diet to a healthier one [1–3]. Afshin et al. (2019) examined the health effects of dietary risks in 195 countries and concluded that globally a suboptimal diet is responsible for more deaths than any other risks, including tobacco smoking [3]. Dietary risks affect people regardless of sex, age, and sociodemographics [3]. One of the (behavioural) dietary factors that are suggested to be improved is the intake of whole-grains [3, 4], with one of the largest gaps between current and optimal whole-grain intake found in Western Europe [3]. One of the whole-grains that is recommended to receive considerably more attention is oats, due to their health and nutritional benefits [5].

Oats are a valuable source of protein, carbohydrates, phytochemicals, vitamins, and minerals, and contain a high content of dietary fibres for a broader range of individuals, including Celiac Disease (CD) patients [5–7]. Oat possesses health benefits against cardiovascular disease (CVD), diabetes, obesity, cancer, and gut microbiome [5, 8, 9], due to several bioactive compounds, such as β-glucan (BG) [5, 8]. The consumption of oat β-glucan (OBG) is associated with lowering the total serum and low-density lipoprotein (LDL) cholesterol levels [10] and decreasing the risk of CVD and diabetes type II [11]. The European Commission approved five health claims, including reduction or maintenance of blood cholesterol by OBG (EU 432/2012) [10, 12]. Oat-based products, such as oat protein concentrate, offer several environmental benefits as a plant-based protein source, including the reduction of diet-related greenhouse gas emissions [13]. In cultivation, oats contribute positively health through their extensive root system, which can positively affect biodiversity [14].

**Fig. 1:**
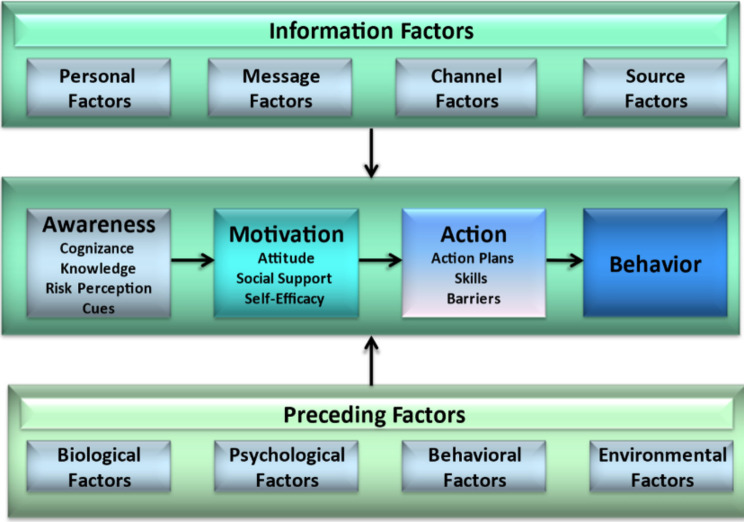
The integrated change model [21]

Limited information is available concerning oat consumption in the Netherlands. Based on whole-grain consumption and the current low dietary fibre intake, it is likely that oat consumption by adults may be low [15, 16]. Developing tailored health communication messages to promote a healthy diet that includes the consumption of oats requires targeted government and marketing strategies. For this, a better understanding of the sociodemographic and motivational factors associated with oat consumption among Dutch adults was sought by using the I-Change Model that addresses these sets of factors (see Fig. 1) [21].

## The Integrated Change Model

The I-Change model integrates various models for understanding health behaviour [17], such as the Health Belief Model (HBM) [18], the Social Cognitive Theory (SCT) [19], and the Theory of Planned Behaviour (TPB) [20]. The socio-cognitive ecological Integrated Change Model [21] has been used successfully to understand and promote healthy lifestyles [22–24]. The ICM suggests three main phases in the behaviour change process: a pre-motivational, motivational, and post-motivational phase that is also known as awareness, motivation, and action (see Fig. 1) [21, 25].

The ICM postulates that a person’s level of awareness is determined by four factors: knowledge, cognizance, risk perceptions, and cues to action. Consumers may be unaware of the health benefits of whole-grains or incorrectly identify refined products for whole-grain sources (e.g., multigrain baking mix) [26–28]. Cognizance is defined as the awareness of someone’s level of a particular behaviour and should be considered carefully as people misperceive their behaviour. People often overestimate their level of healthy eating (e.g., by believing they ate a sufficient amount of vegetables) and thus perceive no need for change [29, 30]. Health-conscious (HC) people have higher levels of knowledge and cognizance [31] and are more likely to have healthy dietary habits [32, 33]. Cues to action make a person aware of a problem and its potential prevention [34]. These cues, which can be internal (like dietary issues) or external (such as marketing), enhance the likelihood of adopting desired behaviours [35, 36]. Risk perceptions refer to the perceived severity and susceptibility to encounter (health) problems, important for motivating healthy behaviours [37].

Once aware of a problem a person needs to become motivated to change a certain risk behaviour. The motivation to adopt a healthy behaviour (also referred to as a person’s intention) is determined by three factors: attitude, social influence beliefs and self-efficacy [21, 38]. Attitude is formed by both rational and emotional beliefs about behaviour or product [39]. A positive attitude towards a product lead to a higher intention to adopt the food product [40, 41]. High dietary fibre content, favourable health outcomes, contribution to satiety, and higher nutritional content are important attitude beliefs for refined-grain products [27, 42]. A lack of perceiving such health benefits is an important disadvantage, whereas their intention increased if they felt convinced about the actual (health) benefits [26, 43, 44]. Various beliefs determine food acceptance attitude, and taste is often a more important factor than health benefits [26, 27, 41, 42, 45–47]. Consumers experiencing positive social support and social norms are more likely to display and change their behaviour [48–50], including areas such as diet quality and whole-grain intake [51, 52]. Self-efficacy, a person’s belief about their capability of performing a specific behaviour in a specific situation, also influences people’s dietary behaviour [44]. People with a high degree of self-efficacy are more likely to adopt healthy foods [26, 43, 44].

Translations of intentions require specific plans and actions. Post-motivational factors that operate in the last phase relate to a person’s actions (action planning, plan enactment, and skills) together with (environmental) barriers. By understanding these phases and factors the ICM helps researchers to recognize the gap between intention and behaviour [53, 54].

The pre-motivational, motivational, and action factors are influenced by various preceding factors (e.g., socio-demographic factors) and information factors (e.g., campaigns promoting oats). Studies have revealed that whole-grain consumption is more common among older adults and those with a high socio-economic background and education [55–57].

However, the interpretation of previous findings concerning oat consumption is hindered by the fact that studies often do not use a comprehensive model nor address oat consumption. Hence, the first objective of this study is to assess which socio-demographic and belief factors are associated with oat consumption in Dutch adults using the ICM. The second objective is to assess how well the chosen model explains both the intention and behaviour to consume oats. This in-depth information may help to create marketing, intervention, and product development strategies that increase oat consumption among adults in the Netherlands.

## Method

### Study design

A cross-sectional study was conducted in accordance with the ICM to address intention and behaviour. First, a qualitative study was carried out to systematically identify and understand the primary beliefs and attitudes towards oat consumption within the target population. We analysed and categorized the data to identify the most salient beliefs mentioned. Next, we compared these results with findings from existing literature on oat consumption, which led to our quantitative questionnaire. We used an online questionnaire hosted on Qualtrics for this quantitative method. The questionnaire was piloted for content and comprehension by the target group and experts on oat consumption and health communication. Participants from the pilot phase were excluded from participation in the quantitative study. Participants were informed about the objectives of the study, and their voluntary participation and were invited to fill in an informed consent form. Responses were treated anonymously and confidentially. The study received ethical approval from the Faculty Research Ethics Committee (FHML-REC) at Maastricht University (FHML-REC/2023/057).

### Participants and recruitment

Following the using Somehagen et al. [58] and using the computer software program G*power (version 3.1) as a frame of reference revealed that at least 269 participants were needed. For the parameter settings, a F-test ANOVA: fixed effects, special, main effects, and interactions were selected using an effect size of 0.25 and a power of 0.8. Correcting for a potential 20% drop-out due to i.e., questionnaire length [59], this resulted in a total required sample of 323 participants. The average time to complete the questionnaire was 16 min.

In both studies, the sample of participants included individuals aged > 18, of both genders, residents in the Netherlands without an allergy to oats, and who read or speak fluently Dutch or English. For the qualitative study, participants were recruited via the researchers’ networks. The participants (*N* = 17) responded to semi-structured discussion interviews with open-ended questions asking about the perceived beliefs associated with oats consumption. These responses revealed the most salient beliefs associated with oats consumption which were then used as items in the quantitative questionnaire. As saturation was met we decided not to include more participants. Participants for the quantitative study were recruited via professional networks, social media channels (e.g., WhatsApp and LinkedIn) and online exchange survey services (e.g., SurveySwap, SurveyCircle). The recruitment process yielded 399 respondents, of which 94 respondents were excluded because they filled out less than 90% of the questions [60], and excluded 6 for not fulfilling age criteria, resulting in a final sample of 299 respondents. The average time to complete the questionnaire was 16 min.

### Measurements

The quantitative questionnaire was developed using a format used by previous studies using the I-Change Model regarding diet consumption [61–63] assessing predisposing factors, motivational factors, and behaviour. For this study this implied that existing scales were used concerning the predisposing factors. To develop a knowledge scale on oat consumption, experts were consulted to identify the most critical items for inclusion. The questions assessing motivational factors were derived from formats previously used in studies employing the I-Change Model. In the context of decision-making research, “Rational Pro” and “Rational Con” refer to the advantages and disadvantages considered when evaluating an option, respectively. “Emotional Pro” and “Emotional Con” refer to the emotional benefits and drawbacks, highlighting how feelings and emotional responses impact decision-making processes. This approach provided preliminary expert validation for the scale used in this descriptive study.

### Predisposing factors and Health consciousness

*Predisposing factors* were assessed using questions from the Dutch Health Monitor [64]. Participants were asked to indicate their age, gender (1 = Male; 2 = Female, 3 = other), and country of origin (1=. Netherlands; 2 = other). Education was merged into four groups (0 = no education, 1 = low education, 2 = middle education, and 3 = high education), working status were merged into two groups (1 = paid work and 2 = no paid work), health problems was merged into two groups (0 = no health problems and 1 = health problems), living status was merged into four groups (1 = lives alone, 2 = lives with parents, 3 = lives with own family, 4 = lives with roommates. Following a specific diet was merged into four groups (1 = Gluten-free, 2 = Diet low in carbohydrates, 3 = Other, 4 = No diet) (see Table 1 for more details).

*Health consciousness* (α = 0.77) was assessed by four items on a 5-point Likert scale (-2 = I strongly disagree; 2 = I strongly agree), e.g., ‘I think a lot about my health’. Items were based on previous research that illustrated the health-conscious consumer [31, 33].

### Awareness factors

*Cognizance* (α = 0.78) (e.g., level of awareness of oat consumption) was assessed by five items on a 5-point Likert scale (-2 = strongly disagree; 2 = strongly agree). *Knowledge* about oats (e.g., their health benefits) was assessed by 10 items (0 = false, 1 = true; ) to measure participants’ knowledge of oats. The scale served as an index, as it measured multiple dimensions (α = 0.41). *Risk perception* was assessed by three items targeting *risk severity* (α = 0.74) and three items targeting participants’ *susceptibility (*α = 0.56) following formats used by previous studies using the ICM Model. Risk severity was assessed on a 5-point Likert scale (0 = not bad at all; 4 = very bad), e.g., ‘How bad would you feel if you got a cardiovascular disease ?’. Risk susceptibility was assessed on a 5-point Likert scale (0 = very low; 4 = very high), e.g., ‘indicate how big the chance is for you to get cardiovascular disease’. Due to insufficient reliability one item (‘Indicate how big the chance is that global warming will increase) was removed, resulting in a reliable susceptibility scale (α = 0.84). *Perceived cues* (α = 0.73) were assessed by 11 statements on a 5-point Likert scale (-2 = strongly disagree; 2 = strongly agree), which measured if and how information was received from their environment, e.g., ‘I have seen/read/heard information about the importance of oats in the daily diet’.

### Motivational factors

*Attitude*,* social support*,* social norm*, and *intention* were measured on a 5-point Likert scale (-2 = strongly disagree; 2 = strongly agree). The *attitude* was assessed by both rational and emotional outcomes that started with ‘if I eat oats daily…’, e.g., ‘I consume a fibre-rich product’. Potential benefits were examined by nine rational (Rational Pro; α = 0.83) and six emotional statements (Emotional Pro; α = 0.79). Potential disadvantages were examined by eight rational (Rational Con; α = 0.78) and seven emotional statements (Emotional Con; α = 0.86). *Social influence* (α = 0.86) was assessed by four items targeting *social support* and four items targeting *social norms and* the impact of (best) friends, partners, family, and colleagues/classmates. The social support and norm items were combined into one scale. *Self-efficacy* (α = 0.78) was assessed by 16 items on a 5-point Likert scale (-2 = very difficult; 2 = very easy), e.g., ‘It is very easy/difficult for me to consume oats daily if…’, e.g., ‘health benefits only manifest in the long term’. *Intention* (α = 0.91) was measured by 12 items on a 5-point Likert scale (-2 = strongly disagree; 2 = strongly agree), e.g., ‘I plan to consume oats at least once a week’.

### Action factors

*Preparatory planning* (α = 0.88) and *action planning* (α = 0.91) were measured on a 5-point Likert scale (-2 = I strongly disagree; 2 = I strongly agree). *Preparatory planning* was measured by seven statements, e.g., ‘I will look for information about oats via the internet’. *Action planning* was measured by 11 items, e.g., ‘I am going to check if I am currently meeting my daily fibre intake’. Participants reported their *behaviour* on a 6-point scale, indicating their typical oat consumption over the past 12 months. The scale ranged from consuming less than 30 g of oats per day (1 tablespoon = 10 g), through consuming 30–70 g per day, to consuming more than 70 g per day, consuming oats weekly, consuming oats monthly, and not consuming oats at all.

### Data analysis

Results were analysed using IBM SPSS Statistics 26 software. Participants were divided into three groups: non-oats consumers (N; *n* = 79; 26.4%), monthly or weekly oats consumers (M; *n* = 109; 36.5%), and daily oats consumers (D; *n* = 111; 37,1%). One-way ANOVAs were used to assess groups’ differences in means. Bonferroni’s post-hoc tests outlined which groups significantly differed. A Chi-square test was used to assess categorical data and a Fisher’s exact test when expected frequencies in a group were less than five. A Pearson’s test assessed correlations between the dependent (reported oat consumption) and independent variables (r_s_ > 0.50 is as strong; 0.30 is moderate, and < 0. is a weak correlation) [65, 66]. A non-hierarchical multiple linear regression analysis estimated which factors were uniquely associated with oat consumption. Assumptions concerning linearity, independence, homoscedasticity, normality, and multicollinearity were met. Results were regarded as significant if *p* < 0.05. Levene’s test was performed to test the homogeneity of variance and corrected when homogeneity was violated.

## Results

### Respondent characteristics and differences

Table 1 displays all the respondents predisposing characteristics. The total sample consisted of significantly more female respondents than men and their average age was 30.5 years. Overall, respondents reported having higher education, being mostly born in the Netherlands, and having no identified health problems or specific diets. Women reported to consume oats more frequently than men.

**Table 1:** Differences in predisposing factors between non-oats consumers (N), weekly or monthly oats consumers (M), and daily oats consumers (D)

	Total		Consumption behaviour							Statistics					
	(n = 299)		N (n = 79)			M (n = 109)		D (n = 111)		χ2/F	df	p	Post-Hoc		
**Age** ^**1**^ (mean ± SD)	30.48 ± 12.72		31.03 ± 13.26		30.37 ± 12.05			30.21 ± 13.08		0.10	2	0.90	-		
**Gender** ^**2,3**^	%	n	%	n	%		n	%	n						
Male	35.1	105	12.7	38	12.0		36	10.4	31	10.15	4	**0.02**			n/a*
Female	64.2	192	13.4	40	24.4		73	26.4	79						
Other	0.7	2	0.3	1	0.0		0	0.3	1						
**Country of origin** (% NL)	76.3	228	20.4	61	27.1		81	28.8	86	0.36	2	0.83			n/a
**Education**^4^ (mean ± SD)	2.64 ± 0.61		2.61 ± 0.61		2.65 ± 0.58			2.67 ± 0.64		0.22	2	0.80		-	
**Paid work** ^**2**^	%	n	%	n	%		N	%	n						
Yes	58.2	174	16.1	48	19.4		58	22.7	68	1.75	2	0.44			n/a
No	41.8	125	10.4	31	17.1		51	14.4	43						
**Household** ^2^	%	n	%	n	%		n	%	n						
Lives alone	19.4	58	4.3	13	7.4		22	7.7	23	9.56	6	0.14			n/a
Lives with parents	27.1	81	10.0	30	8.4		25	8.7	26						
Lives with own family	35.5	106	9.4	28	12.4		37	13.7	41						
Lives with roommates	18.1	54	2.7	8	8.4		25	7.0	21						
**Health problem** identified^2^	%	n	%	n	%		n	%	n						
Yes	28.4	85	7.0	21	12.4		37	9.0	27	2.68	2	0.26			n/a
No	71.6	214	19.4	58	24.1		72	28.1	84						
**Diet** ^2,3^	%	n	%	n	%		n	%	n						
Gluten-free	10.7	32	2.3	7	4.3		13	4.0	12	6.67	6	0.32			n/a
A Diet low in carbohydrates	5.0	15	1.7	5	0.7		2	2.7	8						
Other	9.4	28	1.3	4	4.3		13	3.7	11						
No	74.9	224	21.1	63	27.1		81	26.8	80						

^1^Analysis of variance was performed for all numerical predisposing factors.

^2^Chi-square tests were performed for all categorical predisposing factors.

^3^Fisher’ exact tests were performed when the expected frequencies were less than 5

^4^Mean ± SD of coded educational level (0 = no education, 1 = lower education, 2 = middle education, 3 = High education).

^*^ Not applicable

### Differences in health consciousness and awareness factors

Table 2 shows that non-oats consumers reported significantly less thinking about their health than monthly, weekly, or daily oats consumers (*p* < 0.05, as indicated by bolded values in the table).

**Table 2:** Variance analysis scores of non-oats consumers (N), weekly or monthly oats consumers (M), and daily oats consumers (D) on health consciousness and awareness factors

	Total (*n* = 299)		Mean (SD)			η^2^	F	*p*	Post-Hoc
		N (n = 79)		M (n = 109)	D (n = 111)				
Health consciousness^1^	0.62 (0.72)	0.34 (0.69)		0.68 (0.76)	0.77 (0.65)	0.06	9.27	**0.00**	N < M, D
Cognizance^1^	-0.13 (0.82)	-0.67 (0.66)		-0.17 (0.81)	0.29 (0.70)	0.10	39.94	**0.00**	N < M < D
Knowledge^2^	0.75 (0.16)	0.69 (0.15)		0.77 (0.14)	0.77 (0.18)	0.09	7.02	**0.00**	N < M, D
Riskseverity^3^	3.24 (0.68)	3.00 (0.70)		3.36 (0.58)	3.30 (0.71)	0.17	7.16	**0.00**	N < M, D
Riskssusceptibility^4^	2.31 (0.64)	2.18 (0.64)		2.45 (0.69)	2.26 (0.57)	0.13	4.85	**0.00**	N < M
Cues to action^1^	0.11 (0.63)	-0.26 (0.55)		0.25 (0.63)	0.24 (0.58)	0.33	21.29	**0.00**	N < M, D

^1^ -2 = strongly disagree, -1 = disagree, 0 = neither disagree nor agree, 1 = agree, 2 = strongly agree

^2^ANOVA variance test performed on sum-score knowledge

^3^Perceived severity 0 = not bad at all; 4 = very bad

^4^Perceived susceptibility 0 = very low; 4 = very high

Oat consumers reported a higher level of cognizance than non-oats consumers (Table 2). However, daily oats consumers showed to be significantly more cognizant of their daily oat consumption, the fulfilment of the recommendation of daily dietary fibre intake, and believed that they consumed enough oats daily than the other two groups (Table S1).

Non-oats consumers generally had significantly less knowledge than the other two groups about the fact that eating oats lowers blood cholesterol, may reduce the risk of cardiovascular diseases, and that oat-based dairy substitutes are more environmentally friendly (Table S2).

Table S2 shows that non-oats consumers perceived significantly less risk severity than the other two groups. Monthly or weekly oats consumers were more convinced of the chance of getting cardiovascular diseases than non-oats consumers. Further, monthly, or weekly oats consumers were significantly more convinced of the chance of getting a high blood cholesterol level than the other two groups (Table S3).

Non-oats consumers reported fewer cues prompting oat consumption and cues about new oat products than the other groups. All groups reported few cues of being advised by a doctor/dietician to consume oats because of their health benefits (Table S3).

### Differences in motivational factors

Table 3 shows that non-oats consumers were significantly less convinced of the rational and emotional advantages of daily oat consumption (*p *< 0.05, as indicated by bolded values in the table). No significant differences were found between monthly or weekly oats consumers and daily oats consumers on the attitude items. In-depth analysis showed that non-oats consumers were significantly less convinced that oats being an energy-rich product, that boosts their immune system, will lower cholesterol levels, improve weight control, save costs, and be environmentally friendly than the other two groups (Table S4).

Non-oats consumers were less convinced of the emotional advantages of daily oat consumption than the other two groups. Further, they significantly believed less in the nutritional properties and health- and environmental benefits of daily oat consumption than the other two groups. Non-oats consumers expressed significantly more negative feelings toward daily oat consumption than the other two groups (Table 3 and Table S4). They were significantly more likely than the other two groups to believe that if they consumed oats daily, they would have high expenses, be restricted in their diet, suffer from bloating, consume too many carbohydrates, miss important nutrients, gain weight, and have a spike in their blood sugar (Table S4). Interestingly, all groups addressed that they needed to combine something with oats to make it tastier.

**Table 3:** Variance analysis scores of non-oats consumers (N), weekly or monthly oats consumers (M), and daily oats consumers (D) on motivational and post-motivational factors

	Total (n = 299)		Mean (SD)			η^2^	F	*p*	Post-Hoc
		N (n = 79)		M (n = 109)	D (n = 111)				
Attitude – Rational Pro^1^	0.69 (0.52)	0.44 (0.48)		0.80 (0.49)	0.76 (0.54)	0.08	13.25	**0.00**	N < M, D
Attitude- Emotional Pro^1^	0.29 (0.62)	-0.14 (0.54)		0.40 (0.59)	0.50 (0.56)	0.18	31.89	**0.00**	N < M, D
Attitude- Rational Con^1^	-0.12 (0.62)	0.09 (0.51)		-0.17 (0.64)	-0.22 (0.63)	0.04	6.60	**0.00**	N < M, D
Attitude- Emotional Con^1^	-0.47 (0.75)	-0.02 (0.59)		-0.57 (0.76)	-0.69 (0.70)	0.14	23.31	**0.00**	N < M, D
Social Support	0.45 (0.79)	0.18 (0.90)		0.53 (0.77)	0.56 (0.67)	0.04	6.34	**0.00**	N < M, D
Social Norm^1^	-0.76 (0.89)	-1.19 (0.76)		-0.67 (0.92)	-0.55 (0.84)	0.09	14.03	**0.00**	N < M, D
Self-efficacy	0.19 (0.41)	-0.04 (0.43)		0.23 (0.36)	0.32 (0.37)	0.12	20.59	**0.00**	N < M, D
Intention	0.11 (0.78)	-0.61 (0.77)		0.35 (0.58)	0.39 (0.62)	0.30	65.03	**0.00**	N < M, D
Preparation Planning^1^	-0.19 (0.83)	-0.76 (0.80)		0.08 (0.78)	-0.05 (0.71)	0.17	30.69	**0.00**	N < M, D
Action planning^1^	-0.30 (0.81)	-0.97 (0.75)		-0.06 (0.69)	-0.06 (0.68)	0.24	47.77	**0.00**	N < M, D

^1^-2 = strongly disagree, -1 = disagree, 0 = neither disagree nor agree, 1 = agree, 2 = strongly agree

^2^ANOVA variance test performed on sum-score knowledge

^3^ Perceived severity 0 = not bad at all; 4 = very bad

^4^Perceived susceptibility 0 = very low; 4 = very high

The in-depth analysis revealed that non-oats consumers significantly received less social support and social norms from friends, best friends/partners, and families. In addition, they also experienced fewer social norms from colleagues/friends.

Non-oats consumers had lower levels of self-efficacy towards daily oat consumption compared to the other two groups. In-depth analysis showed that they perceived more difficulties to eat oats in a various settings, such as when health benefits are not directly evident, when costs are high, when they are unaware of the benefits, when they are busy, and when preparation is time-consuming. All groups reported finding it difficult to consume oats daily with a low variety of products, poor taste and texture compared to other grain products, and lack of support from others. They all agreed that it would be easier to consume oats if attention to oats increased and if policymakers recognized their importance (Table S1).

Non-oats consumers had a significantly lower intention on all the intention statements than the other two groups. They only had the intention to consume oats when they had more knowledge about them. Additionally, oats consumers made more preparation and action plans than non-oats consumers (apart from their plans to look for information about oats through diet magazines) (Table S5).

### Explaining the variance using the ICM

As intention is regarded to be the most proximal predicator of behaviour a multiple non-hierarchical linear regression (method forward) was performed with intention as the dependent variable. No multicollinearity was found on the independent variables, the scatter plots showed that the dependent variable was linearly related to all the independent ones. The analysis involved three steps. No significant effect was found with entering predisposing factors.

Different models were used to test the influences of preceding factors (model 1), awareness factors (model 2), and motivational factors (model 3) (Table 4). No significant influence was found regarding predisposing factors. The final model revealed that 55% of the variance for intention could be explained by self-efficacy pros emotional, social support/norms, cons emotional, cues to action, and pro rational as factors with unique association with intention. Cognizance, knowledge, and risk severity did influence intentions as well, but their influences were fully mediated by these motivational factors.

**Table 4:** Multiple non-hierarchical forward linear regression with intention as the dependent variable

	b	SE b	β	*p*	
**Model 1: No variables were entered into the model**					
**Model 2:**					
Cues to action	0.37	0.07	0.30	0.00	
Cognizance	0.20	0.05	0.21	0.00	
Knowledge	0.76	0.25	0.16	0.00	
Risk severity	0.12	0.06	0.10	0.047	
**Model summary**: F = 29.81; R^2^ = 0.29; ΔR^2^ = 0.01; Adjusted R^2^ = 0.28; *p* = 0.00					
**Model 3**:					**Correlation coefficients** ^**1**^
Pro emotional	0.27	0.07	0.21	0.00	0.61^*^
Self-efficacy	0.46	0.10	0.24	0.00	0.59^*^
Social influence	0.24	0.05	0.20	0.00	0.52^*^
Con emotional	-0.14	0.04	-0.13	0.00	-0.40^*^
Cues to action	0.16	0.06	0.13	0.01	0.46^*^
Pro rational	0.17	0.08	0.11	0.03	0.53^*^
**Model summary**: F = 59.34; R^2^ = 0.55; ΔR^2^ = 0.01; Adjusted R^2^ = 0.54; *p* = 0.00					

^1^Pearson’s correlation coefficients of the independent variables with intention

^*^Correlation is significant

A forward linear regression was conducted to determine key influencers of daily oat consumption (Table 5). Multicollinearity was found between intention and preparation (*r* = 0.75; p = < 0.01), intention and action planning (*r* = 0.82; p = < 0.01), and between preparation with action planning (*r* = 0.79; p = < 0.01.

In the first model, predisposing factors indicated oat consumption was associated with being female. The second model showed high cognizance, mediating the gender effect. The third model introduced motivational factors, revealing that emotional disadvantages negatively, and cognizance and emotional advantages positively, affected oat consumption. The fourth model included intention, which positively influenced oat consumption and mediated the impact of emotional advantages. Emotional disadvantages continued to show a negative association. The final model, incorporating preparation and action planning, confirmed these relationships, explaining 38% of the variance in oat consumption behaviours, with a follow-up showing consistent findings.

**Table 5:** Multiple non-hierarchical forward linear regression with behaviour as the dependent variable

	B	SE b	β	*p*	
**Model 1:**					
Gender	0.50	0.18	0.16	0.00	
**Model summary**: F = 7.91; R^2^ = 0.03; ΔR^2^ = 0.03; Adjusted R^2^ = 0.02; *p* = 0.00					
**Model 2**:					
Cognizance	0.93	0.09	0.51	0.00	
**Model summary**: F = 104.58; R^2^ = 0.26; ΔR^2^ = 0.26; Adjusted R^2^ = 0.26; *p* = 0.00					
**Model 3**:					
Cognizance	0.69	0.10	0.38	0.00	
Con emotional	-0.39	0.10	-0.19	0.00	
Pro emotional	0.44	0.13	0.18	0.00	
**Model summary** F = 49.88; R^2^ = 0.34; ΔR^2^ = 0.02; Adjusted R^2^ = 0.33; *p* = 0.00					
**Model 4**:					**Correlation coefficients** ^**1**^
Cognizance	0.63	0.93	0.34	0.00	0.51^*^
Intention	0.58	0.10	0.30	0.00	0.50^*^
Con emotional	-0.30	0.10	-0.15	0.00	-0.37^*^
**Model summary**: F = 60.10; R^2^ = 0.38; ΔR^2^ = 0.02; Adjusted R^2^ = 0.37; *p* = 0.00					
**Model 5: No different variables were entered into the model**					

^1^Pearson’s correlation coefficients of the independent variables with the outcome variable consumption behaviour

^*^Correlation is significant

## Discussion

This study assessed which socio-demographic and belief factors were associated with oat consumption in Dutch Adults. Women reported consuming oats more frequently than men, which corresponds with previous studies regarding whole-grain consumption differences between genders [56, 67]. Other socio-demographic factors did not affect oat consumption, whereas various studies showed that highly educated individuals reported a healthier diet, including a higher whole-grain intake [55–57]. Yet, our sample was relatively highly educated and relatively small.

Oat consumers were more health-conscious and had higher levels of knowledge and cognizance, which corresponded with prior studies [31, 32, 68]. Other studies also demonstrated that low cognizance may hinder the development of motivation and actions to improve behaviour [69]. Lack of knowledge on nutrition and health was a frequently reported barrier by adults for whole-grain consumption [4, 26–28, 70]. This study shows that non-oat consumers had a higher intention to consume oats when they were more knowledgeable. These results strengthen the need for strategies aimed at providing knowledge. Moreover, previous research revealed that the release of dietary guidelines and related media attention increased the availability and sales of whole-grain products [71].

Oat consumers were more convinced of the severity of several health diseases and reported a higher risk susceptibility, supporting the findings of other studies [37, 72]. Non-oats consumers reported perceiving significantly fewer cues to action, supporting findings from previous research showing that such individuals were less likely to adopt such behaviours, especially new or less familiar behaviours [34, 73, 74].

Non-oats consumers perceived fewer advantages from consuming oats daily and perceived the disadvantages more strongly, e.g., higher costs. All groups indicated that to consume oats they need to combine something with oats to make it tastier, supporting the importance of taste, as also found in other studies regarding whole-grain consumption indicating that bad taste and texture, lack of varieties, and high costs are frequently reported barriers [26, 27, 41, 42, 45, 46, 75]. The eating habit was mentioned as an important barrier to whole-grain consumption by non-oats consumers, as also found in other studies [41, 47]. However, when becoming used to eating new food, people are less likely to report time, as a different taste preference has been established [76]. Our study findings also show frequent oat consumers favoured the emotional and rational benefits over the emotional and rational disadvantages.

Non-oats consumers had low social support and unfavourable norms towards oat consumption. Hence, messages aimed at changing the social norm towards oat consumption may be needed as individuals who experience social support are more likely to adopt these behaviours [48–50]. However, all groups scored negatively on the social norms of others, indicating the need for addressing these norms in society. Regarding self-efficacy, all groups found it difficult to consume oats daily if the taste and texture were poor, and if making food with oats is time-consuming. Previous studies mentioned taste, variety of products, and costs toward whole-grain consumption as critical factors for consumer attitudes [4, 26, 45]. This illustrates the need for a variety of new tasty and affordable oat products that can be prepared with little time [77, 78].

Our results further show that non-oats consumers were significantly less engaged in preparation and action plans related to oat consumption. These findings are in line with the theoretical background of the ICM [21] which is understandable, as one will not make plans if not motivated.

The second objective was to identify the factors that are uniquely associated with the intention and the behaviour. For intention these factors were self-efficacy pros emotional, social support/norms, cons emotional, cues to action, and pro-rational. Furthermore, the results support the assumption that the awareness factors also influenced intention but were mediated by the motivational factors [21]. The final model explained 55% of the variance in intention, which is comparable to that found in other studies for eating behaviour [79, 80]. Cues to action were included in both models 2 and 3, classified primarily as an awareness factor within the Integrated Change Model (ICM). Their repeated appearance suggests they may also play a motivational role, potentially impacting behaviour change strategies. This dual functionality invites further theoretical exploration to understand their comprehensive influence on behavioural intentions. Future research could significantly benefit from detailed investigation into how cues to action simultaneously affect awareness and motivation, enriching the theoretical framework of the ICM.

Concerning behaviour, the results show that intention, cognizance, and cons emotional had a significant association with oat consumption, while cognizance had the strongest which illustrates its important role for behaviour. The final model could explain 38% of the variance in consumption, which is also similar to other studies concerning health behaviour [79]. The roles of cognizance and emotional disadvantages in our study may suggest complex processes that shape intentions and actions. Cognizance consistently appeared in all models, highlighting potentially a more fundamental influence on behaviour, whereas the emotional cons also suggest the importance of emotional outcomes. The role of these factors warrants further in-depth study using longitudinal designs.

### Limitations

As with all studies using self-report data, the results may be subject to social desirability [81], and to participant bias due to our recruitment method resulting in more highly educated individuals and more females. Longitudinal studies are also needed to assess factors predicting oat consumption and should have a larger sample size to be able to detect potential subgroup differences (e.g., due to differences in socio-economic background [82]. This is one of the first studies to use a social-cognitive approach to explore oat consumption. Consequently, the development of our questionnaire was challenging due to the scarcity of relevant previous research. Future developments of the questionnaire may require additional elicitation methods to identify cultural and population-specific beliefs about oat consumption. While the questionnaire included multiple items to ensure thorough measurement and potential item reduction, the absence of attention check items might have influenced the accuracy of responses due to possible participant fatigue. Furthermore, we had to limit the number of items to avoid overburdening of respondents and thus drop-out. Consequently, our assessment of the various predisposing factors did not use more elaborate and validated questionnaires. In our study, the categorization of working status and other variables was primarily driven by our research objectives and the necessity to manage data complexity effectively while ensuring meaningful group distinctions. We aimed to strike a balance between the granularity of the data and the practicality of analysis, choosing broader categories to enhance statistical power and interpretability. However, we acknowledge that this approach may reduce the representation of diversity within each group. Additionally, the reliability of the susceptibility scale was insufficient, but was included in the regression model as an index to analyse how well the total model could explain intentions and behaviour. An improved scale for susceptibility is thus recommended for future studies. Furthermore, as no existing scales were available for assessing the motivational factors, we had to develop these scales using input from the literature and validating this with output received from experts and the target group using qualitative information. Future validation studies may be needed to study construct validation of these constructs in greater depth. As cue to actions, cognizance and emotional cons played a more substantial role than anticipated according the assumptions of the I-Change Model, further research is recommended to confirm this pattern.

Despite these limitations, this is the first study, to the author’s knowledge, to depict adult oat consumption behaviour using an integrated model. The results presented relatively good internal validity, as most results were consistent with previous research on whole-grain consumption and the models explained a similar result of variance as other studies.

## Conclusion

This study provides meaningful insights into the facilitators and determinants that affect adults’ oat consumption. This study explained using the ICM 55% of the variance of intention and 38% of the variance in oat consumption. To promote oat consumption, awareness concerning oat consumption needs to increase by fostering cognizance, knowledge, risk perceptions and cues addressing the importance of oat consumption. Actions are needed to realize more positive attitudes towards oat consumption by focusing on both positive rational and emotional outcomes (and correcting some misperceptions regarding the negative outcomes), to create a more supportive social environment (including positive social norms) and to increase their self-efficacy. The dissemination of (health and nutritional) information is a crucial task that can be taken up on a policy level and in a broad partnership by different stakeholders, for instance governments, food producers, and health professionals (e.g., Nationale Aanpak Productverbetering [83]).

## Electronic supplementary material

Below is the link to the electronic supplementary material.

Supplementary Material 1

## Data Availability

The datasets used and/or analysed during the current study are available from the corresponding author upon reasonable request.

## Abbreviations

ICM: The Integrated Change Model
CD: Celiac Disease
CVD: Cardiovascular Disease
BG: β-glucan
OBG: Oat β-glucan
LDL: Low-Density Lipoprotein
GHG: Greenhouse Gas
HBM: Health Belief Model
SCT: Social Cognitive Theory
TPB: Theory of Planned Behaviour
HC: Health Consciousness
N: Non-oats consumers
M: Monthly or weekly oats consumers
D: Daily oats consumers
SD: Standard deviation
