# Supplementary material for: To eat or not to eat oats: factors associated with oats consumption using the I-Change model
Source: BMC Public Health. 2024 Nov 19;24:3215. doi: 10.1186/s12889-024-20044-4 (PMC11577757; doi:10.1186/s12889-024-20044-4)
Supplement: Supplementary file 1 — Supplementary Material 1 [file 12889_2024_20044_MOESM1_ESM.docx]

# Supplementary data

# A: Qualitative Questionnaire

1. Do you consider your whole grain intake and what do you think about the recommendations to consume more whole grain products per day?
2. Thinking about your own whole grain intake, what do you think about the feasibility of this goal?
3. Why do you think whole grain products are encouraged?
4. Are you aware that a high intake of whole grains reduces the risk of cardiovascular diseases?
5. Are you familiar with oats?
6. What oat products do you know?
7. What do you know about oats (regarding health, cultivation, etc.)?
8. What would be the main benefits for you to consume oats?
9. What would be the main disadvantages for you to consume oats?
10. What are personally the factors that make it easy/difficult for you to consume oats?
11. Have you already come across information about oats? Through a dietitian, supermarket, online/offline platforms
12. Which factors would influence your choice to consume oats? Social influences (family, friends, parents, partners): who would support you and who would not? Who eats oats?
13. Are you willing to start consuming oats and if so, as what kind of product?

The following questions provide a general overview of the research population:

1. What is your gender?
2. What is your age in years?
3. What is your country of birth?
4. What is your highest educational attainment?
5. Do you have an allergy to gluten or oats?
6. Do you follow a gluten-free diet?
7. What is your family composition?

# B: Post-hoc comparison on items of health consciousness and cognizance.

*Table S1: Variance analysis scores* *of non-oats consumers (N), weekly or monthly oats consumers (M), and daily oats consumers (D) on health consciousness and cognizance.*

|  |  |  | **Mean (SD)** | |  | **η^2^** | **F** | ***p*** | **Post-Hoc** |
| --- | --- | --- | --- | --- | --- | --- | --- | --- | --- |
|  | **Total**  **(n=299)** | **N**  **(n=79)** | | **M**  **(n=109)** | **D**  **(n=111)** |  |  |  |  |
| **Health consciousness^1^** | | | | | | | | | |
| I think a lot about my health | 0.98 (0.77) | 0.77 (0.88) | | 1.06 (0.70) | 1.06 (0.72) | 0.03 | 4.17 | **0.02** | N < M, D |
| I do my best to live every day as healthy as possible | 0.54 (0.91) | 0.18 (0.84) | | 0.65 (0.92) | 0.69 (0.88) | 0.06 | 9.17 | **0.00** | N < M, D |
| I adjust my diet to live as healthy as possible^2^ | 0.59 (0.9) | 0.15 (0.96) | | 0.72 (0.87) | 0.78 (0.78) | 0.09 | 14.08 | **0.00** | N < M, D |
| I exercise a lot to maintain my health | 0.38 (1.13) | 0.25 (1.13) | | 0.30 (1.21) | 0.54 (1.03) | 0.01 | 1.89 | 0.15 | - |

| **Cognizance^1^** | | | | | | | | |
| --- | --- | --- | --- | --- | --- | --- | --- | --- |
| I am aware of how much whole grain I eat per day | -0.15 (1.23) | -0.71 (1.23) | -0.15 (1.18) | 0.23 (1.12) | 0.09 | 14.90 | **0.00** | N < M, D |
| I am aware of how much oats I eat per day^2^ | -0.29 (1.27) | -1.05 (1.04) | -0.35 (1.16) | 0.30 (1.24) | 0.17 | 31.39 | **0.00** | N < M < D |
| I follow the recommendations of the daily amount of fiber^2^ | -0.25 (1.15) | -0.76 (1.08) | -0.26 (1.20) | 0.12 (1.02) | 0.09 | 14.55 | **0.00** | N < M < D |
| I eat enough whole-grain products every day^2^ | 0.34 (0.96) | 0.04 (0.99) | 0.29 (1.01) | 0.59 (0.80) | 0.05 | 8.39 | **0.00** | N < D |
| I eat enough oats every day | -0.28 (0.98) | -0.86 (0.90) | -0.39 (0.87) | 0.23 (0.87) | 0.20 | 36.36 | **0.00** | N < M < D |
| Note: ‘N,M,and D’ are abbreviations of *non-oats consumers (N), weekly or monthly oats consumers (M), and daily oats consumers (D).*  ^1^ -2 = strongly disagree, -1 = disagree, 0 = neither disagree nor agree, 1 = agree, 2 = strongly agree.  ^2^ Welch’s test was performed as Leven’s test showed homogeneity of variance <0.05. | | | | | | | | |

# Post-hoc comparison of items knowledge

*Table S2: Scores of variance analysis scores of non-oats consumers (N), weekly or monthly oats consumers (M), and daily oats consumers (D) on knowledge.*

|  | **Mean (SD)** | | | | **F** | **p** | **Post-hoc** |
| --- | --- | --- | --- | --- | --- | --- | --- |
|  | **Total**  **(n=299)** | **N**  **(n=79)** | **M**  **(n=109)** | **D**  **(n=111)** |  |  |  |
|  |  |  |  |  |  |  |  |
| Oats are defined as whole grains (true) | 0.79 (0.41) | 0.76 (0.43) | 0.82 (0.39) | 0.79 (0.41) | 0.45 | 0.64 | - |
| Oats are fiber-rich (true) ^1^ | 0.98 (0.15) | 0.99 (0.11) | 0.99 (0.10) | 0.95 (0.21) | 1.82 | 0.16 | - |
| Eating oats lowers blood cholesterol levels (true) ^1^ | 0.79 (0.41) | 0.68 (0.47) | 0.83 (0.37) | 0.82 (0.39) | 3.70 | **0.03** | N < M |
| Oats may reduce the risk of glaucoma (false) ^1^ | 0.40 (0.49) | 0.46 (0.50) | 0.31 (0.46) | 0.44 (0.50) | 2.69 | 0.07 | - |
| Eating oats can reduce the risk of cardiovascular disease (true) ^1^ | 0.81 (0.40) | 0.70 (0.46) | 0.83 (0.37) | 0.86 (0.35) | 4.30 | **0.01** | N < D |
| Oats do not fit in the gluten-free diet (false) ^1^ | 0.53 (0.50) | 0.43 (0.50) | 0.62 (0.49) | 0.50 (0.50) | 3.70 | **0.03** | N < M |
| The cultivation of oats is small in the Netherlands compared to the cultivation of wheat (true) ^1^ | 0.72 (0.45) | 0.65 (0.48) | 0.72 (0.45) | 0.77 (0.42) | 1.92 | 0.15 | - |
| Cultivation of oats provides benefits for soil quality (true) ^1^ | 0.72 (0.45) | 0.65 (0.48) | 0.77 (0.42) | 0.72 (0.45) | 1.78 | 0.17 | - |
| Dairy substitutes have been made based on oats (true) ^1^ | 0.90 (0.30) | 0.84 (0.37) | 0.94 (0.25) | 0.90 (0.30) | 2.52 | 0.08 | - |
| Dairy substitutes based on oats are more environmentally friendly than dairy products (true) ^1^ | 0.85 (0.36) | 0.76 (0.43) | 0.85 (0.36) | 0.89 (0.31) | 3.16 | **0.04** | N < D |
| Note: ‘N,M,and D’ are abbreviations of *non-oats consumers (N), weekly or monthly oats consumers (M), and daily oats consumers (D).*  ^1^ Welch’s test was performed as Levene’s test showed homogeneity of variance <0.05. | | | | | | | |

# Post-hoc comparison on items risk perception and cues to action

*Table S3: Variance analysis scores of non-oats consumers (N), weekly or monthly oats consumers (M), and daily oats consumers (D) on risk perception and cues to action.*

|  |  |  | **Mean (SD)** |  | **η^2^** | **F** | ***p*** | **Post-Hoc** |
| --- | --- | --- | --- | --- | --- | --- | --- | --- |
|  | **Total**  **(n=299)** | **N**  **(n=79)** | **M**  **(n=109)** | **D**  **(n=111)** |  |  |  |  |
| **Risk perception** | | | | | | | | |
| *Please indicate how much you mind the following ^1^:* |  |  |  |  |  |  |  |  |
| How bad would you feel if you got a cardiovascular disease? | 3.49 (0.75) | 3.44 (0.83) | 3.58 (0.64) | 3.45 (0.78) | 0.01 | 1.06 | 0.35 | - |
| How bad would you feel if you got a high cholesterol level?^2^ | 3.09 (0.88) | 2.72 (0.90) | 3.26 (0.70) | 3.20 (0.85) | 0.06 | 10.30 | **0.00** | N < M, D |
| How bad is global warming for you? | 3.14 (0.88) | 2.87 (0.97) | 3.28 (0.73) | 3.36 (0.80) | 0.04 | 6.09 | **0.00** | N < M, D |
| *Indicate how big the chance is for you..^3^.* |  |  |  |  |  |  |  |  |
| to get cardiovascular disease | 1.78 (0.94) | 1.54 (0.93) | 1.94 (1.02) | 1.78 (0.85) | 0.03 | 4.02 | **0.02** | N < M |
| to get high cholesterol | 1.80 (0.95) | 1.70 (0.94) | 2.04 (1.05) | 1.63 (0.81) | 0.04 | 5.76 | **0.00** | N, D < M |
| that global warming will increase | 3.35 (0.71) | 3.29 (0.70) | 3.39 (0.65) | 3.37 (0.77) | 0.00 | 0.44 | 0.64 | - |
| **Cues to action^4^** |  |  |  |  |  |  |  |  |
| I have seen/read/heard information about the importance of oats in the daily diet^2^ | -0.26 (1.19) | -1.03 (0.89) | -0.06 (1.22) | 0.09 (1.19) | 0.15 | 26.59 | **0.00** | N < M, D |
| I have seen/read/heard information about how consumption affects the severity of climate change^2^ | 0.76 (1.09) | 0.61 (1.19) | 0.76 (1.15) | 0.86 (0.94) | 0.01 | 1.29 | 0.28 | - |
| I have been advised by a dietitian/doctor to increase the daily oat consumption^2^ | -1.25 (0.93) | -1.62 (0.61) | -1.11 (0.92) | -1.12 (1.05) | 0.06 | 9.17 | **0.00** | N < M, D |
| I have been advised by other people to increase the daily consumption of oats^2^ | -0.98 (1.11) | -1.52 (0.75) | -0.92 (1.08) | -0.66 (1.22) | 0.09 | 15.42 | **0.00** | N < M, D |
| I have seen/read/heard that there are several new products based on oats (such as oat drinks or oatcakes) ^2^ | 0.69 (1.14) | 0.06 (1.28) | 0.89 (0.95) | 0.93 (1.05) | 0.11 | 17.78 | **0.00** | N < M, D |
| I have seen health claims on oat products | -0.24 (1.20) | -0.80 (1.05) | 0.02 (1.22) | -0.11 (1.15) | 0.08 | 12.63 | **0.00** | N < M, D |
| I know someone with type II diabetes | 0.07 (1.47) | -0.05 (1.51) | 0.22 (1.52) | 0.01 (1.40) | 0.01 | 0.92 | 0.40 | - |
| I know someone with a high blood pressure^2^ | 0.69 (1.18) | 0.35 (1.29) | 0.89 (1.07) | 0.73 (1.15) | 0.03 | 4.96 | **0.01** | N < M |
| I know someone with high cholesterol | 0.46 (1.25) | 0.06 (1.31) | 0.70 (1.22) | 0.51 (1.18) | 0.04 | 6.22 | **0.00** | N < M, D |
| I know someone with gluten intolerance (celiac disease) ^2^ | 0.60 (1.39) | 0.52 (1.49) | 0.59 (1.46) | 0.66 (1.25) | 0.00 | 0.23 | 0.79 | - |
| I know someone with intestinal problems | 0.73 (1.23) | 0.57 (1.33) | 0.83 (1.18) | 0.76 (1.21) | 0.01 | 1.02 | 0.36 | - |
| Note: ‘N,M,and D’ are abbreviations of *non-oats consumers (N), weekly or monthly oats consumers (M), and daily oats consumers (D).*  ^1^Perceived severity 0 = not bad at all; 4 = very bad.  ^2^Welch’s test was performed as Levene’s test showed homogeneity of variance <0.05.  ^3^Perceived susceptibility 0 = very low; 4 = very high.  ^4^-2 = strongly disagree, -1 = disagree, 0 = neither disagree nor agree, 1 = agree, 2 = strongly agree. | | | | | | | | |

# Post-hoc comparison on items attitude, social influence, and self-efficacy

*Table S4: Variance analysis scores of non-oats consumers (N), weekly or monthly oats consumers (M), and daily oats consumers (D) on attitude beliefs, social influence, and self-efficacy.*

|  |  | | |  | | | **Mean (SD)** | | |  | | | **η^2^** | | | **F** | | | ***p*** | | | **Post-Hoc** | |
| --- | --- | --- | --- | --- | --- | --- | --- | --- | --- | --- | --- | --- | --- | --- | --- | --- | --- | --- | --- | --- | --- | --- | --- |
|  | **Total**  **(n=299)** | | | **N**  **(n=79)** | | | **M**  **(n=109)** | | | **D**  **(n=111)** | | |  | | |  | | |  | | |  | |
| **Attitude - Rational Pro^1^;** If I eat oats on a daily basis, | | | | | | | | | | | | | | | | | | | | | | | |
| I receive an energy-rich product^2^ | 0.92 (0.68) | | | 0.68 (0.79) | | | 1.07 (0.60) | | | 0.93 (0.61) | | | 0.06 | | | 7.96 | | | **0.00** | | | N < M, D | |
| I consume a fibre-rich product | 1.16 (0.62) | | | 1.05 (0.66) | | | 1.23 (0.60) | | | 1.16 (0.61) | | | 0.01 | | | 1.90 | | | 0.15 | | | - | |
| I take good care of my intestine | 0.92 (0.75) | | | 0.67 (0.86) | | | 1.04 (0.71) | | | 0.98 (0.67) | | | 0.03 | | | 6.25 | | | **0.00** | | | N < M, D | |
| I take good care of my immune system^2^ | 0.74 (0.79) | | | 0.48 (0.89) | | | 0.85 (0.66) | | | 0.82 (0.79) | | | 0.03 | | | 6.16 | | | **0.00** | | | N < M, D | |
| I lower the risk of health problems | 0.71 (0.88) | | | 0.53 (0.87) | | | 0.80 (0.82) | | | 0.75 (0.93) | | | 0.02 | | | 2.28 | | | 0.10 | | | - | |
| I lower the cholesterol level in blood | 0.59 (0.85) | | | 0.32 (0.84) | | | 0.71 (0.83) | | | 0.67 (0.85) | | | 0.03 | | | 5.71 | | | **0.00** | | | N < M, D | |
| I have better control of my weight | 0.35 (0.90) | | | 0.08 (0.94) | | | 0.71 (0.83) | | | 0.67 (0.85) | | | 0.03 | | | 5.34 | | | **0.00** | | | N < M, D | |
| I save costs^2^ | 0.09 (1.02) | | | -0.37 (0.80) | | | 0.21 (1.01) | | | 0.29 (1.08) | | | 0.09 | | | 11.49 | | | **0.00** | | | N < M, D | |
| I choose an environmentally friendly product^2^ | 0.70 (0.74) | | | 0.49 (0.78) | | | 0.82 (0.63) | | | 0.74 (0.78) | | | 0.02 | | | 4.71 | | | **0.01** | | | N < M | |
| **Attitude - Emotional Pro^1^;** If I eat oats on a daily basis, | | | | | | | | | | | | | | | | | | | | | | | |
| I feel healthier | 0.56 (0.81) | | | 0.14 (0.83) | | | 0.72 (0.82) | | | 0.70 (0.69) | | | 0.08 | | | 15.63 | | | **0.00** | | | N < M, D | |
| I enjoy the food | 0.23 (0.92) | | | -0.29 (0.82) | | | 0.32 (0.89) | | | 0.52 (0.87) | | | 0.12 | | | 21.26 | | | **0.00** | | | N < M, D | |
| I feel full faster | 0.61 (0.88) | | | 0.33 (0.75) | | | 0.61 (0.86) | | | 0.80 (0.93) | | | 0.10 | | | 6.98 | | | **0.00** | | | N < D | |
| I feel good | 0.37 (0.84) | | | -0.32 (0.73) | | | 0.50 (0.70) | | | 0.73 (0.75) | | | 0.24 | | | 50.74 | | | **0.00** | | | N < M, D | |
| I am proud of myself, because I eat an environmentally friendly product | 0.11 (0.90) | | | -0.23 (0.86) | | | 0.32 (0.81) | | | 0.15 (0.95) | | | 0.03 | | | 9.06 | | | **0.00** | | | N < M, D | |
| I worry less about my cholesterol level | -0.11 (0.97) | | | -0.46 (0.80) | | | -0.05 (1.08) | | | 0.06 (0.90) | | | 0.05 | | | 7.38 | | | **0.00** | | | N < M, D | |
| **Attitude - Rational Con^1^;** If I eat oats on a daily basis, | | | | | | | | | | | | | | | | | | | | | | | |
| I spend a lot of time preparing the food | -0.21 (1.01) | | | -0.20 (0.92) | | | -0.18 (1.07) | | | -0.25 (1.01) | | | 0.02 | | | 0.13 | | | 0.87 | | | - | |
| I spend a lot of time finding products and recipes | -0.21 (0.98) | | | -0.04 (1.01) | | | -0.23 (0.99) | | | -0.31 (0.96) | | | 0.03 | | | 1.76 | | | 0.17 | | | - | |
| I spend too much money on food | -0.35 (0.96) | | | -0.04 (0.88) | | | -0.50 (0.97) | | | -0.42 (0.97) | | | 0.03 | | | 5.91 | | | **0.00** | | | M, D < N | |
| I find that I limit myself very much in terms of food to few products | -0.08 (1.04) | | | 0.19 (0.92) | | | -0.19 (1.08) | | | -0.18 (1.05) | | | 0.03 | | | 3.69 | | | **0.03** | | | M < N | |
| I don't make it easy on myself because of the necessary preparations | -0.15 (1.02) | | | -0.04 (0.91) | | | -0.19 (1.06) | | | -0.18 (1.05) | | | 0.03 | | | 0.62 | | | 0.54 | | | - | |
| I always have to combine something to make it tastier | 0.67 (0.99) | | | 0.71 (0.95) | | | 0.69 (1.02) | | | 0.61 (0.98) | | | 0.03 | | | 0.26 | | | 0.77 | | | - | |
| it causes me bloating^2^ | -0.33 (0.91) | | | 0.11 (0.78) | | | -0.42 (0.92) | | | -0.57 (0.88) | | | 0.11 | | | 15.04 | | | **0.00** | | | M, D < N | |
| I am consuming too many carbohydrates^2^ | -0.32 (0.95) | | | 0.00 (0.89) | | | -0.38 (0.98) | | | -0.49 (0.90) | | | 0.09 | | | 6.67 | | | **0.00** | | | M, D < N | |
| **Attitude - Emotional Con^1^;** If I eat oats on a daily basis, | | | | | | | | | | | | | | | | | | | | | | | |
| I regret not being able to eat my normal food^2^ | -0.53 (1.06) | | | 0.06 (1.17) | | | -0.67 (1.01) | | | -0.81 (0.86) | | | 0.13 | | | 19.23 | | | **0.00** | | | M, D < N | |
| I do not like the food | -0.40 (0.99) | | | 0.25 (0.87) | | | -0.54 (0.94) | | | -0.72 (0.90) | | | 0.15 | | | 28.88 | | | **0.00** | | | M, D < N | |
| I do not enjoy the food | -0.32 (1.05) | | | 0.18 (0.94) | | | -0.57 (0.95) | | | -0.43 (1.10) | | | 0.07 | | | 13.68 | | | **0.00** | | | M, D < N | |
| I will have a boring food pattern | -0.03 (1.10) | | | 0.49 (0.87) | | | -0.21 (1.15) | | | -0.23 (1.09) | | | 0.06 | | | 13.09 | | | **0.00** | | | M, D < N | |
| I am afraid I will miss other important nutrients because of it^2^ | -0.55 (1.02) | | | -0.14 (1.09) | | | -0.57 (1.01) | | | -0.83 (0.88) | | | 0.07 | | | 11.23 | | | **0.00** | | | M, D < N | |
| I am afraid I will gain weight | -0.69 (0.94) | | | -0.53 (0.87) | | | -0.62 (1.01) | | | -0.86 (0.91) | | | 0.05 | | | 3.33 | | | **0.04** | | | D < N | |
| I am afraid it will cause a spike in my blood sugar level^2^ | -0.77 (0.88) | | | -0.44 (0.89) | | | -0.83 (0.92) | | | -0.94 (0.79) | | | 0.06 | | | 8.03 | | | **0.00** | | | M, D < N | |
| **Social influence:** | | | | | | | | | | | | | | | | | | | | | | | |
| *Social support^1^***:** | | | | | | | | | | | | | | | | | | | | | | | |
| My friends will support the daily consumption of oats | | | 0.40 (0.90) | | | 0.13 (0.98) | | | 0.49 (0.90) | | | 0.50 (0.82) | | | 0.04 | | | 4.98 | | | **0.01** | | N < M, D |
| My best friend/partner will support the daily consumption of oats | | | 0.54 (0.91) | | | 0.27 (0.97) | | | 0.60 (0.94) | | | 0.68 (0.80) | | | 0.03 | | | 5.32 | | | **0.00** | | N < M, D |
| My family will support the daily consumption of oats^2^ | | | 0.47 (0.91) | | | 0.15 (1.03) | | | 0.59 (0.91) | | | 0.59 (0.74) | | | 0.04 | | | 7.11 | | | **0.00** | | N < M, D |
| My colleagues/classmates will support the daily consumption of oats | | | 0.38 (0.87) | | | 0.19 (0.96) | | | 0.44 (0.85) | | | 0.45 (0.81) | | | 0.02 | | | 2.53 | | | 0.08 | | - |
| *Social Norm^1^***:** | | | | | | | | | | | | | | | | | | | | | | | |
| According to my friends, I should eat oats every day^2^ | | | -0.82 (0.93) | | | -1.24 (0.75) | | | -0.80 (0.93) | | | -0.53 (0.94) | | | 0.09 | | | 14.60 | | | **0.00** | | N < M, D |
| According to my best friend/partner, I should eat oats every day^2^ | | | -0.69 (1.04) | | | -1.14 (0.90) | | | -0.52 (1.15) | | | -0.53 (0.92) | | | 0.07 | | | 10.73 | | | **0.00** | | N < M, D |
| According to my family, I should eat oats every day^2^ | | | -0.72 (0.99) | | | -1.16 (0.85) | | | -0.61 (0.99) | | | -0.51 (0.99) | | | 0.06 | | | 11.91 | | | **0.00** | | N < M, D |
| According to my colleagues/classmates, I should eat oats daily^2^ | | | -0.83 (0.95) | | | -1.22 (0.76) | | | -0.76 (0.99) | | | -0.61 (0.95) | | | 0.06 | | | 10.28 | | | **0.00** | | N < M, D |
| **Self-efficacy:** It is very difficult/easy for me to consume oats daily, if^3^ | | | | | | | | | | | | | | | | | | | | | | | |
| the cost is higher than other grain products | | -0.33 (0.89) | | | -0.58 (0.86) | | | -0.29 (0.87) | | | -0.20 (0.91) | | | 0.03 | | | 4.54 | | | **0.01** | | | N < D |
| I have to prepare it differently than my standard products | | -0.13 (0.88) | | | -0.43 (0.89) | | | -0.17 (0.85) | | | 0.12 (0.83) | | | 0.08 | | | 9.74 | | | **0.00** | | | N, M < D |
| there is a more variety of oat products^2^ | | 0.71 (0.78) | | | 0.58 (0.78) | | | 0.67 (0.84) | | | 0.85 (0.70) | | | 0.03 | | | 2.95 | | | 0.05^*^ | | | - |
| the taste and texture are poor compared to other grain products | | -0.56 (0.96) | | | -0.68 (1.04) | | | -0.57 (0.92) | | | -0.45 (0.93) | | | 0.04 | | | 1.38 | | | 0.25 | | | - |
| I combine it with other products (such as yogurt) | | 0.73 (0.84) | | | 0.56 (0.90) | | | 0.80 (0.84) | | | 0.79 (0.78) | | | 0.02 | | | 2.38 | | | 0.09 | | | - |
| it brings health benefits^2^ | | 0.90 (0.74) | | | 0.56 (0.78) | | | 1.08 (0.67) | | | 0.96 (0.71) | | | 0.04 | | | 13.06 | | | **0.00** | | | N < M, D |
| health benefits only manifest in the long term | | 0.29 (0.85) | | | 0.00 (0.89) | | | 0.39 (0.83) | | | 0.41 (0.80) | | | 0.05 | | | 6.69 | | | **0.00** | | | N < M, D |
| I do not know the benefits | | -0.30 (0.91) | | | -0.61 (0.82) | | | -0.29 (0.93) | | | -0.08 (0.89) | | | 0.05 | | | 8.17 | | | **0.00** | | | N < D |
| more attention is paid to oats | | 0.58 (0.75) | | | 0.28 (0.77) | | | 0.71 (0.72) | | | 0.66 (0.71) | | | 0.04 | | | 9.01 | | | **0.00** | | | N < M, D |
| it is promoted on social media | | 0.41 (0.82) | | | 0.14 (0.89) | | | 0.50 (0.76) | | | 0.52 (0.78) | | | 004 | | | 6.28 | | | **0.00** | | | N < M, D |
| policymakers also recognize the importance of oats (such as, with interventions and subsidies) | | 0.59 (0.76) | | | 0.39 (0.74) | | | 0.69 (0.77) | | | 0.64 (0.74) | | | 0.02 | | | 3.93 | | | **0.02** | | | N < M |
| I do not receive support from others (e.g., family members/roommates) | | -0.07 (0.87) | | | -0.18 (0.92) | | | -0.05 (0.93) | | | -0.02 (0.76) | | | 0.02 | | | 0.85 | | | 0.43 | | | - |
| I am very busy | | -0.18 (0.97) | | | -0.49 (0.89) | | | -0.22 (1.01) | | | 0.08 (0.93) | | | 0.06 | | | 8.61 | | | **0.00** | | | N < D |
| making food with oats is time-consuming | | -0.66 (0.87) | | | -0.91 (0.83) | | | -0.64 (0.96) | | | -0.49 (0.75) | | | 0.08 | | | 5.75 | | | **0.00** | | | N < D |
| it is an environmentally friendly product | | 0.68 (0.74) | | | 0.48 (0.71) | | | 0.71 (0.77) | | | 0.79 (0.70) | | | 0.04 | | | 4.31 | | | **0.01** | | | N < D |
| I have health problems (such as intestinal problems) | | 0.43 (0.95) | | | 0.30 (1.08) | | | 0.43 (0.90) | | | 0.51 (0.90) | | | 0.02 | | | 1.12 | | | 0.33 | | | - |
| Note: ‘N,M,and D’ are abbreviations of *non-oats consumers (N), weekly or monthly oats consumers (M), and daily oats consumers (D).*  ^1^ -2 = strongly disagree, -1 = disagree, 0 = neither disagree nor agree, 1 = agree, 2 = strongly agree.  ^2^Welch’s test was performed as Levene’s test showed homogeneity of variance <0.05.  ^3^ -2: very difficult: 2: very easy. | | | | | | | | | | | | | | | | | | | | | | | |

# Post-hoc comparison on items intention, preparation planning and action

*Table S5: Variance analysis scores of non-oats consumers (N), weekly or monthly oats consumers (M), and daily oats consumers (D) on intention, preparation planning, and action.*

|  |  | |  | | **Mean (SD)** | |  | | **η^2^** | | **F** | | ***p*** | | **Post-Hoc** | |
| --- | --- | --- | --- | --- | --- | --- | --- | --- | --- | --- | --- | --- | --- | --- | --- | --- |
|  | **Total**  **(n=299)** | | **N**  **(n=79)** | | **M**  **(n=109)** | | **D**  **(n=111)** | |  | |  | |  | |  | |
| **Intention^1^** | | | | | | | | | | | | | | | | |
| I plan to consume at least 20 grams of oats daily^2^ | -0.30 (1.09) | | -1.09 (0.85) | | -0.27 (0.96) | | 0.22 (1.04) | | 0.22 | | 42.51 | | **0.00** | | N < M < D | |
| I plan to consume oats at least once a week | 0.24 (1.25) | | -0.84 (1.08) | | 0.57 (1.08) | | 0.69 (1.06) | | 0.21 | | 54.66 | | **0.00** | | N < M, D | |
| I am open to consume oats when I have more knowledge about oats^2^ | 0.81 (0.89) | | 0.57 (1.07) | | 0.97 (0.76) | | 0.82 (0.84) | | 0.02 | | 4.78 | | **0.01** | | N < M | |
| I plan to consume more oats when people around me see its importance^2^ | 0.10 (1.12) | | -0.43 (1.26) | | 0.34 (0.95) | | 0.23 (1.05) | | 0.08 | | 13.12 | | **0.00** | | N < M, D | |
| I plan to look for oat recipes | 0.21 (1.09) | | -0.51 (1.11) | | 0.51 (0.98) | | 0.43 (0.95) | | 0.11 | | 27.83 | | **0.00** | | N < M, D | |
| I plan to use oats as a substitute/additive for/to other grains (such as, in a pie and pancakes) | 0.14 (1.11) | | -0.85 (0.91) | | 0.51 (1.02) | | 0.48 (0.87) | | 0.22 | | 59.52 | | **0.00** | | N < M, D | |
| I plan to eat oats when there are more products^2^ | 0.39 (0.97) | | -0.23 (1.12) | | 0.70 (0.79) | | 0.54 (0.83) | | 0.10 | | 26.44 | | **0.00** | | N < M, D | |
| I plan to eat more oats during my breakfast (with e.g., oat flakes/granola, oatmeal and oat bread) ^2^ | 0.33 (1.16) | | -0.71 (1.06) | | 0.67 (0.93) | | 0.74 (0.97) | | 0.23 | | 60.26 | | **0.00** | | N < M, D | |
| I plan to eat more oats during my lunch (e.g., oat bread and oat tortillas) | -0.19 (1.06) | | -0.84 (0.90) | | -0.08 (1.05) | | 0.17 (0.97) | | 0.14 | | 25.27 | | **0.00** | | N < M, D | |
| I plan to eat more oats during my dinner (with e.g., oat tortillas, oat pasta, and roti) | -0.28 (1.05) | | -0.81 (0.92) | | -0.14 (1.08) | | -0.04 (0.98) | | 0.09 | | 15.46 | | **0.00** | | N < M, D | |
| I plan to increase my oat consumption with an oat snack (sweet or savory) | -0.05 (1.08) | | -0.76 (0.98) | | 0.17 (1.05) | | 0.24 (0.96) | | 0.14 | | 27.53 | | **0.00** | | N < M, D | |
| I plan to increase my oat consumption with oat-based dairy substitutes^2^ | -0.07 (1.18) | | -0.80 (0.99) | | 0.20 (1.19) | | 0.18 (1.08) | | 0.10 | | 23.44 | | **0.00** | | N < M, D | |
| **Preparation planning^1^** | | | | | | | | | | | | | | | | |
| I will look for information about the health benefits of daily consumption of oats | | -0.05 (1.09) | | -0.67 (1.08) | | 0.27 (1.04) | | 0.07 (0.96) | | 0.08 | | 20.52 | | **0.00** | | N < M, D |
| I will look for information about the benefits of oats on the environment | | -0.04 (1.12) | | -0.65 (1.06) | | 0.23 (1.13) | | 0.12 (1.00) | | 0.07 | | 17.51 | | **0.00** | | N < M, D |
| I will look for information about oats via the internet^2^ | | 0.18 (1.11) | | -0.28 (1.24) | | 0.40 (1.05) | | 0.28 (0.97) | | 0.07 | | 10.01 | | **0.00** | | N < M, D |
| I will look for information about oats through diet magazines | | -0.90 (1.00) | | -1.11 (0.93) | | -0.77 (1.03) | | -0.86 (0.99) | | 0.01 | | 2.84 | | 0.06 | | - |
| I will look for oat products in the (online) supermarket | | 0.00 (1.13) | | -0.72 (1.01) | | 0.28 (1.09) | | 0.23 (1.03) | | 0.10 | | 25.37 | | **0.00** | | N < M, D |
| I will look for recipes wherein they use oats | | 0.12 (1.13) | | -0.66 (1.07) | | 0.56 (1.03) | | 0.24 (0.99) | | 0.10 | | 33.37 | | **0.00** | | N < M, D |
| I will ask my family, friends and colleagues about the benefits and possibilities of oats^2^ | | -0.63 (1.06) | | -1.22 (0.74) | | -0.41 (1.15) | | -0.43 (1.01) | | 0.08 | | 18.08 | | **0.00** | | N < M, D |
| **Action^1^** | | | | | | | | | | | | | | | | |
| I am going to check if I currently meet the daily amount of whole grains | | -0.27 (1.10) | | -0.89 (1.07) | | -0.04 (1.07) | | -0.05 (0.97) | | 0.11 | | 19.15 | | **0.00** | | N < M, D |
| I am going to check if I am currently meeting my daily fiber intake | | -0.18 (1.11) | | -0.77 (1.14) | | 0.04 (1.08) | | 0.02 (0.97) | | 0.10 | | 16.58 | | **0.00** | | N < M, D |
| I am going to search on the internet what are the options to increase my oat consumption | | -0.23 (1.12) | | -0.80 (1.02) | | 0.01 (1.11) | | -0.07 (1.07) | | 0.06 | | 15.03 | | **0.00** | | N < M, D |
| I am going to buy oats to make oatmeal^2^ | | -0.05 (1.16) | | -0.99 (0.94) | | 0.29 (1.06) | | 0.28 (1.04) | | 0.19 | | 45.33 | | **0.00** | | N < M, D |
| I am going to buy/make oat bread so that I can eat oats daily^2^ | | -0.61 (1.07) | | -1.05 (0.96) | | -0.47 (1.07) | | -0.42 (1.07) | | 0.06 | | 9.90 | | **0.00** | | N < M, D |
| I am going to buy/make oat granola and add it to my breakfast (like, in the yogurt) | | -0.11 (1.12) | | -0.89 (1.04) | | 0.28 (1.01) | | 0.05 (1.02) | | 0.11 | | 32.46 | | **0.00** | | N < M, D |
| I am going to replace my regular snacks with oat snacks | | -0.44 (1.01) | | -1.03 (0.90) | | -0.28 (1.00) | | 0.20 (0.94) | | 0.11 | | 20.06 | | **0.00** | | N < M, D |
| I am going to make the next time pancakes with oats^2^ | | -0.18 (1.19) | | -1.01 (0.90) | | 0.09 (1.16) | | 0.14 (1.13) | | 0.13 | | 31.31 | | **0.00** | | N < M, D |
| I am going to increase my oat consumption by replacing my dairy with oat-based dairy substitutes^2^ | | -0.32 (1.19) | | -1.10 (0.91) | | -0.06 (1.18) | | -0.04 (1.13) | | 0.14 | | 27.04 | | **0.00** | | N < M, D |
| I am going to replace my regular pasta products with oat-based pasta products^2^ | | -0.49 (1.03) | | -1.08 (0.83) | | -0.37 (0.97) | | -0.21 (1.06) | | 0.10 | | 19.96 | | **0.00** | | N < M, D |
| I am going to replace my normal tortillas with oat tortillas | | -0.42 (1.07) | | -1.04 (0.91) | | -0.23 (1.06) | | -0.16 (1.00) | | 0.10 | | 20.70 | | **0.00** | | N < M, D |
| Note: ‘N,M,and D’ are abbreviations of *non-oats consumers (N), weekly or monthly oats consumers (M), and daily oats consumers (D).*  ^1^ -2 = strongly disagree, -1 = disagree, 0 = neither disagree nor agree, 1 = agree, 2 = strongly agree.  ^2^ Welch’s test was performed as Levene’s test showed homogeneity of variance <0.05. | | | | | | | | | | | | | | | | |
